# From taxonomic literature to cybertaxonomic content

**DOI:** 10.1186/1741-7007-10-87

**Published:** 2012-10-31

**Authors:** Jeremy Miller, Torsten Dikow, Donat Agosti, Guido Sautter, Terry Catapano, Lyubomir Penev, Zhi-Qiang Zhang, Dean Pentcheff, Richard Pyle, Stan Blum, Cynthia Parr, Chris Freeland, Tom Garnett, Linda S Ford, Burgert Muller, Leo Smith, Ginger Strader, Teodor Georgiev, Laurence Bénichou

**Affiliations:** 1 Naturalis Biodiversity Center, Department of Terrestrial Zoology, Darwinweg 2, 2333 CR Leiden, The Netherlands; 2 Department of Entomology, California Academy of Sciences, 55 Music Concourse Drive, Golden Gate Park, San Francisco, California, USA; 3 Field Museum of Natural History, Biodiversity Synthesis Center, 1400 South Lake Shore Drive, Chicago, Illinois 60605, USA; 4 Division of Invertebrate Zoology, American Museum of Natural History, Central Park West at 79th Street, New York, New York, 10024, USA; 5 Plazi, Zinggstrasse 16, Bern, Switzerland; 6 Karlsruhe Institute of Technology, Institut für Programmstrukturen und Datenorganisation, Am Fasanengarten 5, Karlsruhe, Germany; 7 Columbia University, Libraries - Digital Programs Division ,New York, New York, USA; 8 Pensoft, ZooKeys Editorial Office, Sofia, Bulgaria; 9 New Zealand Arthropod Collection, Landcare Research, 231 Morrin Road, Auckland, New Zealand; 10 Natural History Museum of Los Angeles County, Crustacea Section, Los Angeles, California, USA; 11 Bernice P. Bishop Museum, Department of Natural Sciences, Honolulu, Hawaii, USA; 12 California Academy of Sciences, Center for Applied Biodiversity Informatics, 55 Music Concourse Drive, Golden Gate Park, San Francisco, California, USA; 13 Encyclopedia of Life, National Museum of Natural History, Smithsonian Institution, Washington, DC, USA; 14 Missouri Botanical Garden, Biodiversity Heritage Library, St. Louis, Missouri, USA; 15 National Museum of Natural History, Smithsonian Institution, Biodiversity Heritage Library, Washington, DC, USA; 16 Harvard University, Museum of Comparative Zoology, 26 Oxford St, Cambridge, Massachusetts, USA; 17 KwaZulu-Natal Museum, Department of Natural Sciences, Pietermaritzburg, South Africa; 18 Field Museum of Natural History, Department of Zoology, Chicago, Illinois, USA; 19 Smithsonian Institution Scholarly Press, Washington, DC, USA; 20 Museum National d'Histoire Naturelle, Paris, France

**Keywords:** cybertaxonomy, open access publishing, semantic content, XML markup

## 

John Perry Barlow wrote song lyrics for the epically touring American rock band The Grateful Dead. The band was known for its eclectic mixture of musical styles, epic live improvisational episodes, and hordes of devoted fans that followed the musicians on tour. Among these fans were the 'tapers', who recorded more than 95% of the Grateful Dead's live shows. In contrast with typical expectations of behavior at live concerts, recording Grateful Dead shows by audience members was not considered inappropriate. On the contrary, it was allowed, even facilitated by the band and their sound crew. The band encouraged exchange and distribution of these tapes, as long as it was purely noncommercial. Inspired by this experience, Barlow went on to articulate an unconventional theory of the economy of information, and how the way we value information is almost diametrically opposed to the way we value physical goods. While the latter is driven by scarcity, information is more valuable when it is more accessible and usable. His argument is encapsulated in the following passages from an article entitled 'Selling Wine without Bottles: The Economy of Mind on the Global Net', which first appeared in Wired in 1993:

*In regard to my own soft product, rock and roll songs, there is no question that the band I write them for, the Grateful Dead, has increased its popularity enormously by giving them away. We have been letting people tape our concerts since the early seventies, but instead of reducing the demand for our product, we are now the largest concert draw in America, a fact which is at least in part attributable to the popularity generated by those tapes*.

*With physical goods, there is a direct correlation between scarcity and value. Gold is more valuable than wheat, even though you can't eat it*.

*While this is not always the case, the situation with information is usually precisely the reverse. Most soft goods increase in value as they become more common. Familiarity is an important asset in the world of information. It may often be the case that the best thing you can do to raise the demand for your product is to give it away*.

As scientists, ideas are our business, and information is our product, so recognizing the economy of ideas may help us maximize the value of what we do.

Taxonomy is a fundamental science that provides the scaffolding for biology. But the true value of taxonomic data remains unrealized because basic biodiversity information remains fragmented and unevenly accessible. Taxonomy helps us recognize species and map their distributions by generating text descriptions, images, and records of when and where they have been observed. Current rates of species extinction, habitat loss, and climate change mean that taxonomy has never been more relevant. Biodiversity is one of the most information-rich fields of human knowledge [1], but advances in basic cybertaxonomic infrastructure have only recently provided the tools to organize biodiversity information in ways that respond to a wide range of user groups, including ecologists, land managers, and interested citizens, not to mention the benefits of readily accessible information to the global taxonomic community. The call to revitalize taxonomy by embracing the internet has been sounded for more than a decade [2]. The time is ripe to significantly increase the volume of taxonomic information freely available online. But simply posting information online will not automatically reinvigorate taxonomy. There are myriad online sites dedicated to particular taxa or projects. These are useful to users interested in questions within the site's domains. But the greater potential lies in mechanisms for aggregating primary source data in ways that allow users to filter and recombine data easily and flexibly for whatever purposes they imagine [1,3,4].

## The structure of taxonomic data

Taxonomic papers are generally a synthesis of a limited set of elements, including text descriptions, scientific names and nomenclatural acts, literature references, images, specimen occurrence records, and increasingly DNA sequences. The role of the author is to link specimens (with their associated occurrence records) to nomenclature, express observations and hypotheses as text, and document observations with images and quantities. In traditional taxonomic publishing, all of these elements are merged together into a document. But what if these elements could simultaneously be released and maintained as discrete data tied to the publication? This would have effects both within and beyond the taxonomic community. With access to data elements in electronic form, data consumers (for example, taxonomists, ecologists, conservationists, molecular biologists) could use the publication in more flexible ways. This could aid in the taxonomic utility of the work, facilitate the recognition of new discoveries, and increase the testability (and hence the scientific quality) of the work. The key to unlocking this potential is semantic tagging.

Semantic tagging is a method of assigning markers, or tags, to a text string so the meaning of that string is discoverable and readable by computers [5]. Data elements organized and tagged according to accepted standards are trivial to combine. By contrast, reconciling the content of multiple traditional publications on the same group can require dedicated study and effort. Once in parsed form, data are available for recombination and repurposing. Associating data elements with a publication adds a measure of credibility based on the reputation of the authors, the review process of the publication venue, and the date of publication [6]. This contrasts with, for example, the museum-collections-based data aggregation model that currently dominates GBIF (Global Biodiversity Information Facility, http://data.gbif.org), where data credibility rests with the originating institution. To the extent that the corpus of taxonomic literature can be parsed and aggregated by various cybertaxonomic repositories, those repositories become powerful tools for fundamental information about the state of biodiversity knowledge, meta-analysis, and data reuse for a wide range of applications, including public outreach [1,3,4,7,8].

Once we agree that it would be desirable to have semantically tagged taxonomic data elements digitally interlinked and associated with publications, there are several ways to approach content development. The strategy we focus on is the journal-centered approach, considering both retrospective and prospective content. The retrospective portion involves converting legacy publications from their current formats (for example, print, PDFs) into parsed and digitally distributed content. The prospective portion involves semantic tagging embedded during the editorial production process. It seems unrealistic for the large number of individual taxonomists, each publishing a handful of taxonomic papers per year, to keep current with the technology. But publishers of journals with an emphasis in taxonomy are better positioned to develop and maintain efficient and current processes.

**Figure 1:**
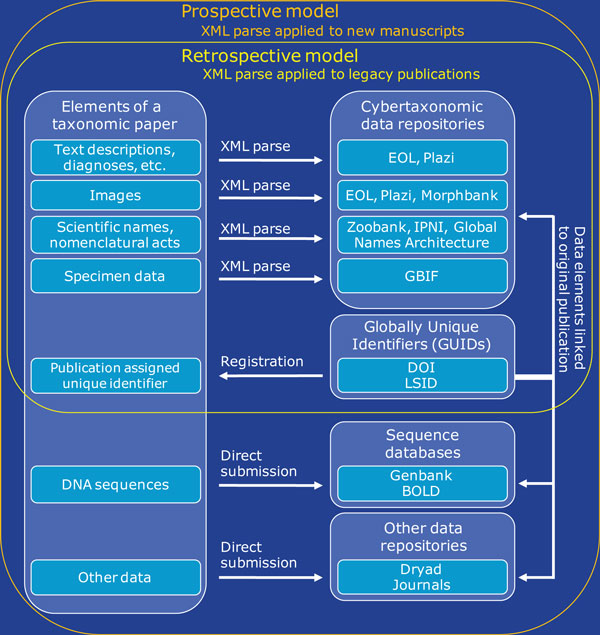
**Schematic diagram of data elements found in taxonomic publications and exemplar cybertaxonomic resources appropriate to hosting each data class**. Semantic tagging of text elements, images, taxonomic nomenclature, and specimen data can be applied retrospectively to legacy publications using tools such as GoldenGATE (http://plazi.org/?q=GoldenGATE). Tagging can also be part of the prospective production process for new taxonomic manuscripts. Some electronic data elements in new papers (for example, DNA sequences) are currently deposited in online repositories by authors. A registered GUID (globally unique identifier) included in the metadata of all electronic data sets links derivative datasets back to the original source publication. BOLD, Barcode of Life Database (http://www.barcodinglife.com/); DOI, Digital Object Identifier (http://www.doi.org/); Dryad (http://datadryad.org/); EOL, Encyclopedia of Life (http://www.eol.org/); GBIF, Global Biodiversity Information Facility (http://data.gbif.org); GenBank (http://www.ncbi.nlm.nih.gov/genbank/); Global Names Architecture (http://globalnames.org/); LSID, Life Science Identifier, Morphbank (http://www.morphbank.net); IPNI, International Plant Names Index (http://www.ipni.org/); Plazi (http://plazi.org/); XML, Extensible Markup Language (http://www.w3.org/XML/); ZooBank (http://www.zoobank.org/).

The infrastructure is already in place for cybertaxonomic resources to recognize and aggregate content appropriate to their focus from documents semantically tagged according to XML standards [5]. We see a future where major taxonomic journals routinely expose new and legacy content in ways that can be discovered, aggregated, and distributed by a community of cybertaxonomic repositories (Figure 1). The source XML documents might be hosted by individual journals or kept in a common repository such as Plazi (http://plazi.org/). To facilitate the integration of biological information across diverse sources, each digitized publication should be distinguished by a globally unique identifier (GUID), such as a registered DOI (digital object identifier) or LSID (Life Science Identifier), linking the data elements back to the original source, author, and journal [7].

## The prospective approach

The undisputed leader in prospective parsing and dissemination of taxonomic content is Pensoft (http://www.pensoft.net/), publisher of cutting edge open access cybertaxonomy journals, including *ZooKeys*, *PhytoKeys*, and *MycoKeys*. The first of these, *ZooKeys*, started with a revolutionary publishing model designed to disseminate biodiversity data in both traditional and innovative ways. This included registering all new nomenclatural acts with the ZooBank database (http://www.zoobank.org/) and providing species descriptions to the Encyclopedia of Life (http://www.eol.org/) as a routine part of their work flow. Pensoft adopted Plazi's TaxPub XML schema as a starting point [5]. Over time, *ZooKeys *and her sister journals continued to push the envelope of cybertaxonomic publishing, constantly looking for new avenues for sharing biodiversity data. It has become clear that parsing content into semantically tagged elements and publishing primary data in standardized digital form are an efficient and powerful combination for repurposing content from primary source taxonomic literature [5,8]. Open access is essential to facilitate the flow of data from taxonomic literature to cybertaxonomic repositories. The many benefits of open access in taxonomic publishing have been convincingly enumerated elsewhere [9,10]. They certainly include improving accessibility of publications internationally and beyond the immediate taxonomic community.

We would encourage more taxonomic journals to adopt some of Pensoft's routine practices. For prospective publishing, there are similarities between document layout and XML markup that might be combined to mitigate any added burden. However, the costs and benefits of this approach will depend largely on the volume of taxonomic papers published by a particular journal or publisher. Based on experience with Plazi and Pensoft, XML markup multiplies production costs by 5 and takes 0.5 to 2 minutes per page. It may not make sense for journals to provide XML markup if covering a range of topics that only occasionally includes taxonomy. In such cases, responsibility for XML markup may be better placed with the author. A new generation of tools is starting to appear that allow authors to semanticize content while writing a manuscript. These include the Publication Module in EDIT's Scratchpads (http://scratchpads.eu/) [11] and the Pensoft Writing Tool (PWT; http://www.pensoft.net/services-for-journals).

## The retrospective approach

**Figure 2:**
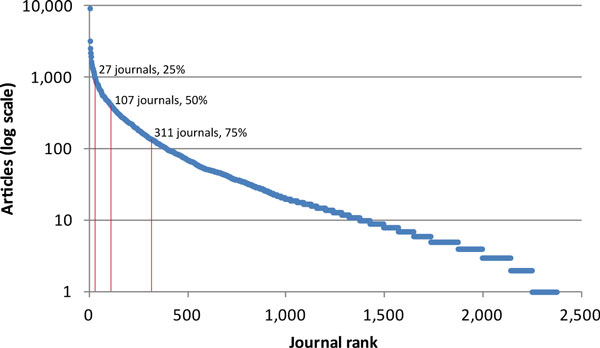
**All articles in Zoological Record (1864 to 2012) found with the Systematics search terms 'Revision' or 'New taxa' or 'Diagnosis' or 'Description' or 'Taxonomy' sorted by journal**. Search returns nearly 200,000 articles but results are strongly concentrated by journal. So XML markup of the legacy content of the top 27, 107, and 311 ranked journals would respectively cover 25, 50, and 75% of all taxonomic articles in Zoology. Progress toward marking up the long tail of articles published in journals with relatively few taxonomic papers each would be approached by a distributed network of self-motivated individuals using the next generation of markup software.

Although thousands of journals have published taxonomic papers, just a few are responsible for the vast majority. Focusing resources on marking up the legacy content of major taxonomic journals is an efficient way to move a large body of content online. Addressing the long tail of taxonomic content in smaller journals will require a more distributed approach (Figure 2).

The GoldenGATE XML Markup Editor (free download from http://plazi.org/?q=GoldenGATE) is an innovative and powerful tool for converting taxonomic papers into XML documents. GoldenGATE analyses the PDF of a taxonomic paper and automatically recognizes the semantic meaning of the various elements. A human operator then checks and refines the markup. Because journals tend to follow particular formatting conventions, GoldenGATE can be optimized for accuracy with a particular journal - one reason to focus effort by journal, rather than by taxon for example. The result is an XML document based on the TaxonX schema (http://www.taxonx.org/). This document can then be made available to cybertaxonomic data aggregators. In its current form, GoldenGATE requires study, training (estimated 2 to 3 days), and practice before an operator can be considered proficient. As the value of marked up legacy content from major journals becomes more widely appreciated, developers will be incentivized to create a new generation of software tools tailored for a more distributed user community. Taxonomists will start filling in the gaps by marking up and sharing articles relevant to their own work.

Taxonomic materials that have not been published in open access journals are not excluded from retrospective semantic markup [12]. Under a limited set of circumstances, 'Fair Use' clauses of copyright law permit copying protected materials without consulting the copyright holder (but see [3]). However, data mining of copyrighted materials remains controversial and is opposed by some publishers [13].

## Conclusions

The journal-centric approach to cybertaxonomy should be understood to mean that we are not advocating any change in the way taxonomists conduct their science. Rather, taxonomists can focus effort and time on their research and on submitting manuscripts to journals that provide traditional peer review and technical editing, but now also make the information available to the community of online cybertaxonomic resources. This will enfranchise new classes of users who can use the fruits of taxonomic research in ways anticipated and unanticipated by the authors. Barlow's economic paradigm teaches us that the more accessible, comprehensive and usable taxonomic information is, the more it will be valued. Therefore, the goal of taxonomy should not simply be to describe every species on Earth, but to make that information accessible.

Understanding biodiversity is such a massive challenge that the traditional approach of working in relative isolation to produce paper publications for colleagues and libraries is simply too inefficient. But there is an alternative model: increase the value of knowledge by giving it away to the world on a massive scale, and empower others to help it grow. More than 250 years after taxonomists began describing and cataloging species, the internet and an ethic of information sharing let us organize biodiversity information in a way that is responsive to the questions and the challenges of our changing world.
